# Single Collateral Reconstructions Reveal Distinct Phases of Corticospinal Remodeling after Spinal Cord Injury

**DOI:** 10.1371/journal.pone.0030461

**Published:** 2012-01-24

**Authors:** Claudia Lang, Xiaoli Guo, Martin Kerschensteiner, Florence M. Bareyre

**Affiliations:** Research Unit Therapy Development, Institute of Clinical Neuroimmunology, Ludwig-Maximilians-Universität München, Munich, Germany; University of Cincinnatti, United States of America

## Abstract

**Background:**

Injuries to the spinal cord often result in severe functional deficits that, in case of incomplete injuries, can be partially compensated by axonal remodeling. The corticospinal tract (CST), for example, responds to a thoracic transection with the formation of an intraspinal detour circuit. The key step for the formation of the detour circuit is the sprouting of new CST collaterals in the cervical spinal cord that contact local interneurons. How individual collaterals are formed and refined over time is incompletely understood.

**Methodology/Principal Findings:**

We traced the hindlimb corticospinal tract at different timepoints after lesion to show that cervical collateral formation is initiated in the first 10 days. These collaterals can then persist for at least 24 weeks. Interestingly, both major and minor CST components contribute to the formation of persistent CST collaterals. We then developed an approach to label single CST collaterals based on viral gene transfer of the *Cre recombinase* to a small number of cortical projection neurons in *Thy1-STP-YFP* or *Thy1*-*Brainbow* mice. Reconstruction and analysis of single collaterals for up to 12 weeks after lesion revealed that CST remodeling evolves in 3 phases. Collateral growth is initiated in the first 10 days after lesion. Between 10 days and 3–4 weeks after lesion elongated and highly branched collaterals form in the gray matter, the complexity of which depends on the CST component they originate from. Finally, between 3–4 weeks and 12 weeks after lesion the size of CST collaterals remains largely unchanged, while the pattern of their contacts onto interneurons matures.

**Conclusions/Significance:**

This study provides a comprehensive anatomical analysis of CST reorganization after injury and reveals that CST remodeling occurs in distinct phases. Our results and techniques should facilitate future efforts to unravel the mechanisms that govern CST remodeling and to promote functional recovery after spinal cord injury.

## Introduction

Injury to the spinal cord leads to a disruption of ascending and descending fiber tracts followed by loss of sensation and voluntary movements below the level of the lesion [1]. Whereas a complete transection of the spinal cord often leads to permanent disabilities, incomplete injuries can be followed by spontaneous functional recovery [2]–[4]. An important anatomical feature underlying this functional recovery is the remodeling of damaged axonal connections [5]–[8]. Many insights into how axons remodel after lesion stems from the study of the corticospinal tract (CST). The CST is a major descending motor pathway that mediates skilled movements in all mammalian species [9], [10]. The CST in rodents consists of a main component that runs at the base of the dorsal funiculus and minor components in the dorso-lateral and ventral funiculus [11]–[13]. In recent years we and others have studied how the hindlimb portion of the CST responds to a thoracic dorsal hemisection. Using a combination of anterograde, retrograde and trans-synaptic tracing techniques we have previously shown that the formation of intraspinal detour circuits are a key component of CST remodeling after injury [6], [14]. Detour circuits are formed in the following steps: First, the lesioned CST fibers sprout new collaterals in the cervical spinal cord above the level of lesion. These collaterals then extend to the intermediate layers of the cervical gray matter. There they form contacts with different populations of spinal interneurons, including long propriospinal neurons, a population of interneurons that are involved in coupling of forelimb and hindlimb movement [15]–[18]. These long propriospinal neurons, the axons of which bypass the lesion in the ventral funiculus, in return increase their projections to hindlimb motoneurons in the lumbar spinal cord. Electrophysiological and detailed behavioral and kinematic analysis show that this and similar detour circuits play a key role for the recovery of CST function [6], [7].

While it is thus established that the formation of CST collaterals is a key step of axonal remodeling after injury, we still know very little about how long these collaterals persist, from which CST components they originate and how their complexity and projection pattern evolves over time. Analysis of mice traced by injection with the anterograde tracer BDA (Biotin Dextran Amine) in the hindlimb motor cortex and perfused at 10 days to 24 weeks after a dorsal hemisection of the mid-thoracic spinal cord now revealed the following findings: CST collaterals primarily started to grow in the first 10 days after injury. Both major and minor CST components contributed to this emergence of collaterals. Once emerged, the majority of CST collaterals persisted at least for up to 24 weeks after lesion. To study how these collaterals evolve over a long period of time (for up to 12 weeks after lesion), we labeled single CST collaterals by viral gene transfer of *Cre recombinase* to a small number of cortical projection neurons in *Thy1-Stp-YFP* [13] and *Thy1-Brainbow mice* [19]. The reconstruction of single collaterals emerging from main and minor CST components showed that dramatic changes in collateral length and complexity occur between 10 days and 4 weeks after injury. These parameters remain largely stable between 4 weeks and 12 weeks after lesion. Analysis of the CST contacts onto interneurons however indicated that while the morphology of the collaterals remained largely unchanged during the late stage of the remodeling process, their synaptic projections were still refined. We can further show that while the overall timing of the remodeling is similar in main and minor CST collaterals their individual complexity differed depending on their origin. Taken together our result suggest that CST remodeling after SCI occurs in 3 subsequent phases: a growth initiation phase (within the first 10 days after injury), which is followed by a collateral formation phase (between 10 days and 3–4 weeks after injury) and a later maturation phase (between 3–4 weeks and 12 weeks after injury).

## Results

### Cervical CST collaterals primarily emerge in the first 10 days after lesion and persist over time

**Figure 1:**
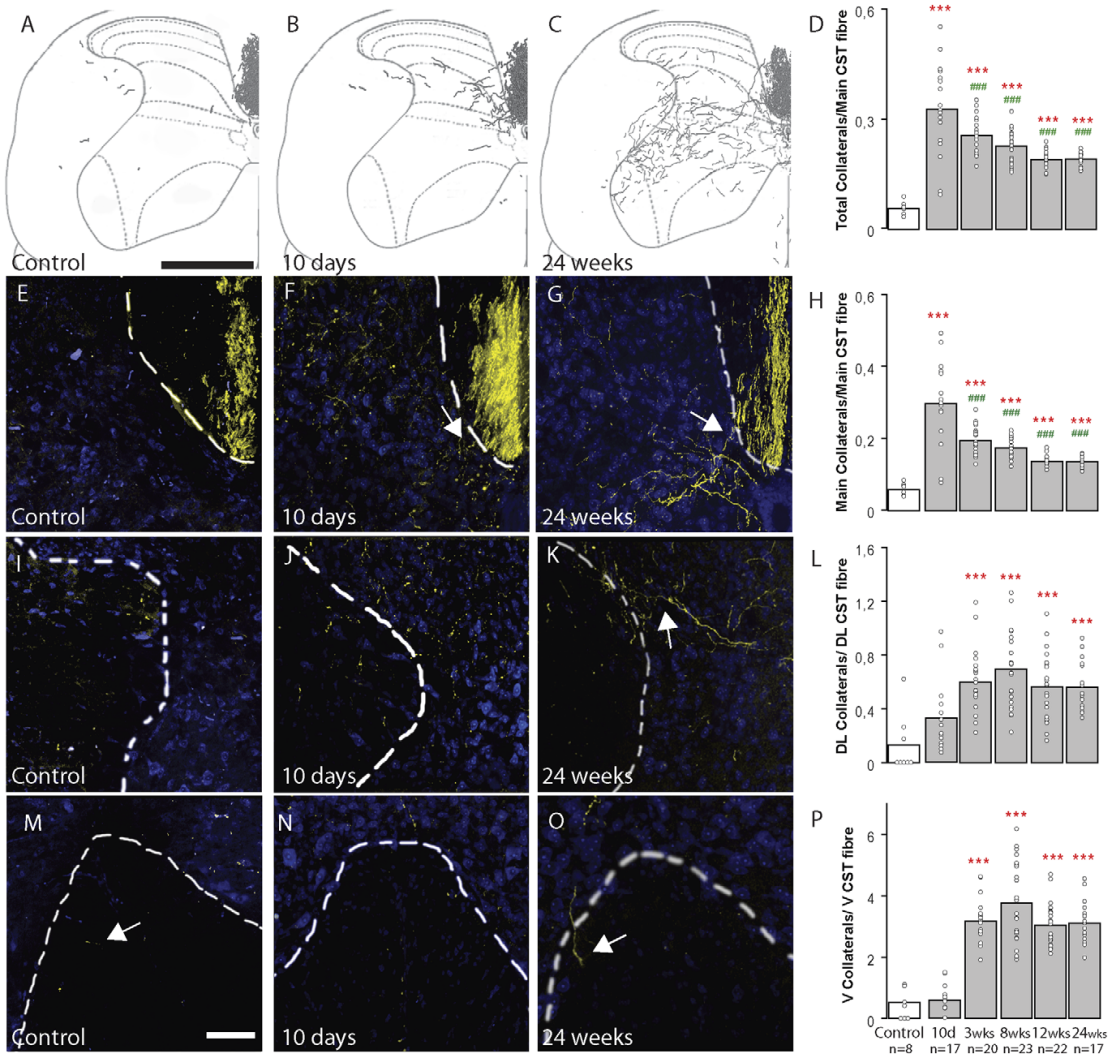
Population analysis of hindlimb CST collateral formation at different timepoints after SCI. (A–C) Reconstruction of hindlimb CST collaterals (black) from 5 consecutive sections in the cervical spinal cord of control mice (A) and of mice perfused 10 days (B) and 24 weeks (C) following SCI. (D) Quantification of the total numbers of collaterals emerging from all CST components in the cervical gray matter of control mice and of mice at different timepoints following SCI. (E–G) Confocal images of main CST (BDA, yellow) and the adjacent gray matter (Neurotrace, blue; border shown by dashed white line) in control mice (E) and in mice perfused 10 days (F, arrow indicates CST collateral emerging from main CST) and 24 weeks (G, arrow indicates CST collateral emerging from main CST) following SCI. (H) Quantification of the number of collaterals emerging from the main CST component at different timepoints following SCI. (I–K) Confocal images of the minor dorso-lateral CST (BDA, yellow) and the adjacent gray matter (Neurotrace, blue; border shown by dashed white line) in control mice (I) and in mice perfused 10 days (J) and 24 weeks (K, arrow indicates CST collateral emerging from dorso-lateral CST) following SCI. (L) Quantification of the number of collaterals emerging from the minor dorso-lateral CST component at different timepoints following SCI. (M–O) Confocal images of the minor ventral CST (BDA, yellow) and the adjacent gray matter (Neurotrace, blue; border shown by dashed white line) in control mice (M, arrow indicates ventral CST fiber) and in mice perfused 10 days (N) and 24 weeks (O, arrow indicates collateral emerging from ventral CST) following SCI. (P) Quantification of the number of collaterals emerging from the minor ventral CST component at different timepoints following SCI. Asterisks indicate significance compared to the unlesioned controls. Pound signs indicate significance compared to the 10-day timepoint. Scale bar in A (also for B,C), 500 µm; in M (also for E–O), 100 µm.

Injection of BDA 10,000 into the hindlimb motor cortex revealed three components of the hindlimb CST in the spinal cord (**Fig. 1A**). The main CST component runs at the base of the dorsal funiculus and contains 97.6±0.27% (n = 8 mice) of labeled CST fibers. The minor CST components run in the dorso-lateral and ventral funiculus and contain 2.1±0.23% (n = 8 mice) and 0.3±0.04% (n = 8 mice) of labeled CST fibers, respectively.

In unlesioned adult mice, axons arising from all hindlimb CST components sent only very few collaterals into the gray matter of the cervical spinal cord (level C3–C5, **Fig. 1 A, D**). However, as early as 10 days following a mid-thoracic lesion, the number of CST collaterals in the cervical cord gray matter increased more than 4-fold (**Fig. 1 B, D**). Over the following weeks the number of cervical collaterals slowly decreased. Still the majority of the collaterals persisted long-term and was still detected as late as 24 weeks after injury (**Fig. 1 C, D**). Over this timeframe the collaterals, which in most cases have just started to enter the spinal gray matter at 10 days after lesion (**Fig. 1 B**), extended further and mainly projected to the intermediate layers of the spinal cord (**Fig. 1 C)**. When we analyzed the contribution of different CST components to the formation of cervical collaterals, we found that, while in absolute number most of the collaterals arose from the main CST, the relative number of new collaterals that emerge per labeled fiber was several-fold higher for the minor dorso-lateral and ventral CST components (**Fig. 1 E–P**). Notably, while the number of newly formed CST collaterals emerging from the main CST significantly declined over time (**Fig. 1 H**), the number of collaterals derived from the minor CST components remained stable for the entire observation period (**Fig. 1 L, P**).

### Complex CST collaterals form between 10 days and 4 weeks after lesion

**Figure 2:**
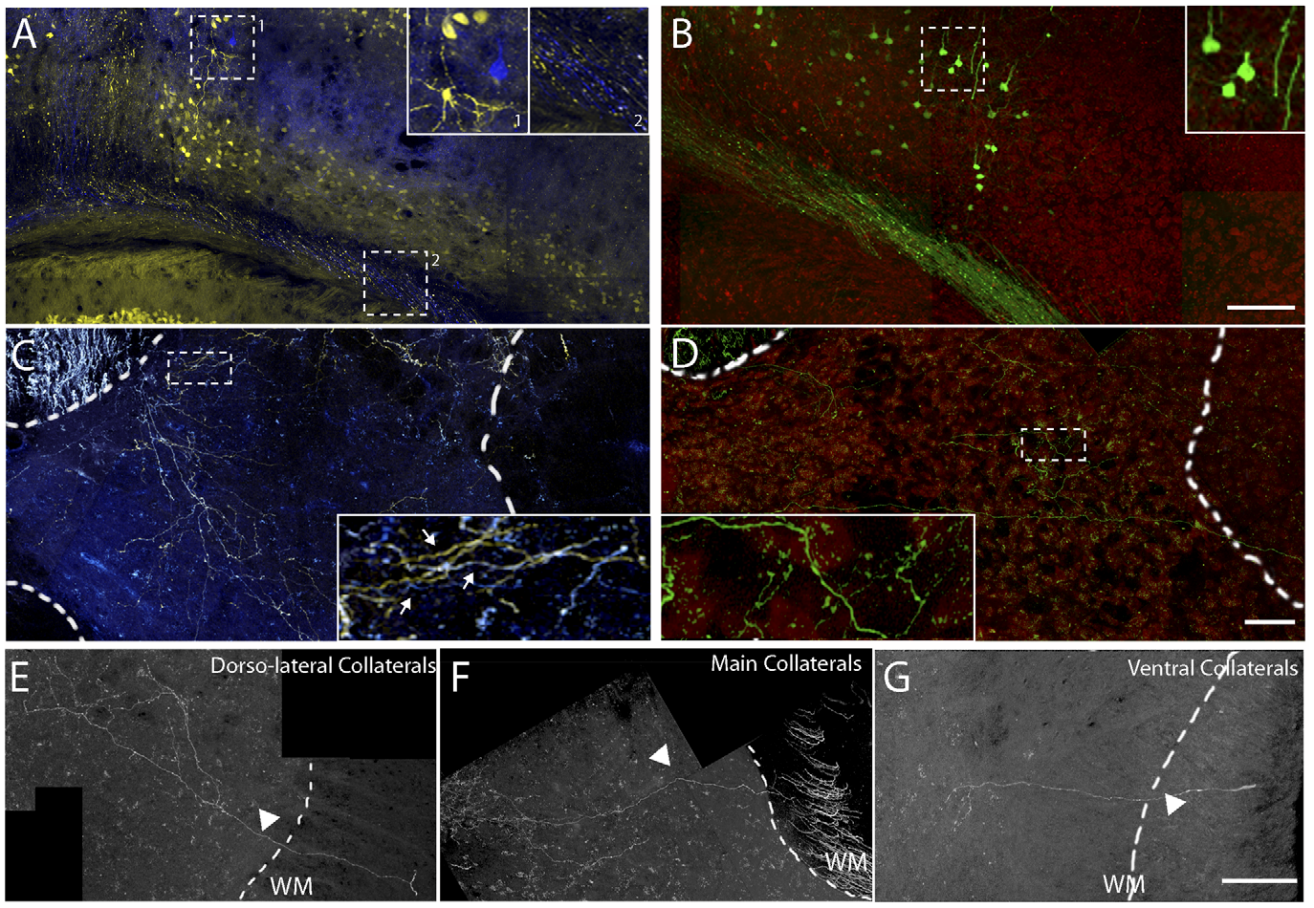
Strategies for labeling individual CST collaterals. (A, B) Confocal images of the cortex of a *Thy1-Brainbow* (A; YFP, yellow; CFP, blue) and *Thy1-Stp-YFP* mouse (B; YFP, green; Neurotrace, red) after local injection of rAAV-Cre. Boxed areas are magnified 2 times in insets. (C,D) Confocal images of CST collaterals in the cervical spinal cord of a *Thy1-Brainbow* (C; YFP, yellow; CFP, blue) and *Thy1-Stp-YFP* (D; YFP, green; Neurotrace, red) mouse after injection of rAAV-Cre in the cortex. Boxed areas are magnified 2 times in insets. Arrows in inset in C indicate different collaterals expressing either CFP (blue), YFP (yellow) or a combination of both (white). Dashed white line indicates the outline of the spinal gray matter. (E–G) Confocal images of individual collaterals (white) emerging from the main CST (F), the dorso-lateral CST (E) and the ventral CST (G) following SCI. Arrows indicate individual collaterals. Dashed white lines indicate the outline of the spinal gray matter. Scale bar in B (also for A),100 µm; Scale bar in D (also for C),100 µm; Scale bar in G (also for E,F), 50 µm.

To label single CST collaterals, we took advantage of *Thy1-Stp-YFP* [13] and *Thy1-Brainbow* [19] mice. In these mouse lines the presence of *Cre recombinase* either starts (in the case of *Thy1-Stp-YFP*) or changes (in the case of *Thy1-Brainbow* mice) the expression of fluorescent proteins in the affected neurons. Expression of *Cre recombinase* was restricted to a small number of cortical projection neurons by stereotactically injecting small amounts of a recombinant Adeno-Associated Virus expressing *Cre recombinase* (rAAV-Cre) into the hindlimb motor cortex (**Fig. 2 A, B**). Single collaterals emerging from the axons of transduced cortical projection neurons could than be identified based on their unique labeling in the cervical spinal cord and reconstructed from serial cross-sections (**Fig. 2 C–G**).

**Figure 3:**
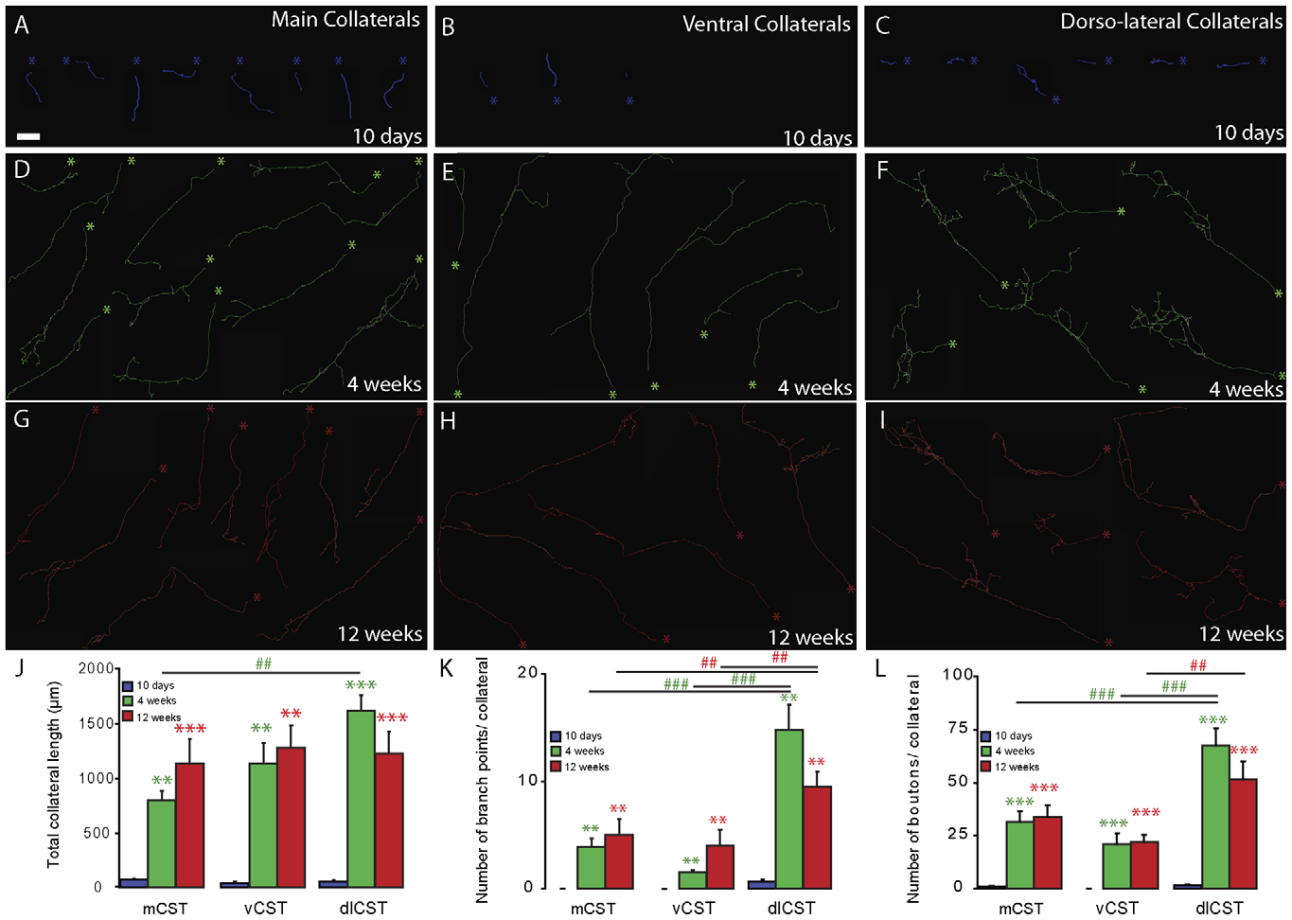
Reconstruction of individual hindlimb CST collaterals at different timepoints after spinal cord injury. (A–C) Reconstruction of individual collaterals (blue asterisks indicate the entry point of the collateral in the gray matter) emerging from the main dorsal (A) and the minor ventral (B) and dorso-lateral (C) CST components at 10 days following SCI. (D–F) Reconstruction of individual collaterals (green) emerging from the main dorsal (D) and the minor ventral (E) and dorso-lateral (F) CST components at 4 weeks following SCI. (G–I) Reconstruction of individual collaterals (red) emerging from the main dorsal (G) and the minor ventral (H) and dorso-lateral (I) CST components at 12 weeks following SCI. (J–L) Quantification of the total collateral length (J), the number of branchpoints/collateral (K) and the number of boutons/collateral (L) measured in individually reconstructed collaterals at different timepoints after SCI. Blue bars, 10-day timepoint; green bars, 3-week timepoint; red bars, 12-week timepoint. Asterisks indicate significant differences compared to the 10-day timepoint. Pound signs indicate significant differences between collaterals emerging from different CST components at 3 weeks (green) and 12 weeks (red) after injury. Scale bar in A (also for B–I), 50 µm.

We used this approach to analyze the structure of cervical collaterals emerging from main and minor CST components at 10 days, 4 weeks and 12 weeks after a mid-thoracic hemisection of the spinal cord (**Fig. 3**). At 10 days following the injury, CST collaterals emerging from all CST components were fairly short (**Fig. 3 A–C, J**), had a simple, mostly unbranched structure (**Fig. 3 K**) and showed very few, if any, boutons (**Fig. 3 L**). At 4 weeks after lesion, the collaterals were substantially longer (**Fig. 3 D–F, J**), had a complex often highly branched structure (**Fig. 3 K**) and a higher number of boutons (**Fig. 3 L**). At this time, the anatomical structure of a collateral depended on its white matter origin. Compared to main CST collaterals, collaterals emerging from the ventral CST were long but showed a relatively simple structure with few branch points and boutons (**Fig. 3 E, J–L**). In contrast, collaterals emerging from the dorso-lateral CST component had a highly complex structure and significantly more branchpoints and boutons compared to both ventral and main CST collaterals (**Fig. 3 J–L**). While the structure of CST collaterals thus evolved substantially between 10 days and 4 weeks after lesion, collaterals emerging from all CST components remain largely unchanged between 4 weeks and 12 weeks after injury (**Fig. 3 G–L**). Consequently, at 12 weeks after lesion dorso-lateral CST collaterals still had significantly more branchpoints than main CST collaterals and more branchpoints and boutons than ventral CST collaterals (**Fig. 3 J–L**).

### Synaptic differentiation of newly formed CST boutons

**Figure 4:**
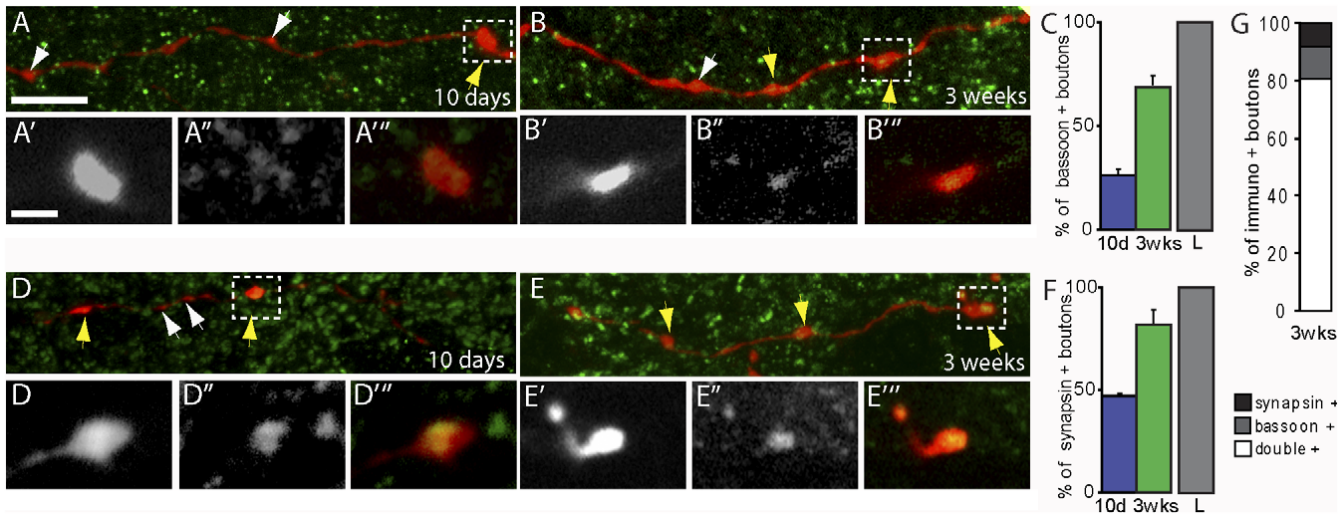
Synaptic differentiation of newly formed CST boutons. (A–B) Confocal images of bassoon immmunostaining (green) in the cervical spinal cord of mice with a traced hindlimb CST (BDA, red) perfused 10 days (A) and 3 weeks following SCI (B). Yellow arrows indicate boutons that were immunoreactive for bassoon, white arrows indicate those that were not. (A′–A′″) Single plane confocal image of the boutons boxed in A showing the collateral (A′; BDA, white), bassoon immunostaining (A″; white) and the overlay (A′″; BDA, red; bassoon, green) at 10 days after SCI. (B′–B′″) Single plane confocal image of the bouton boxed in B showing the collateral (B′; BDA, white), bassoon immunostaining (B″, white) and the overlay (B′″; BDA, red; bassoon, green) at 3 weeks after SCI. (C) Quantification of the number of boutons on hindlimb CST collaterals that were immunopositive for bassoon at 10 days and 3 weeks following SCI in the cervical cord. The percentages were normalized to the expression pattern in the lumbar cord (L) of control animals (which was set to 100%). (D–E) Confocal images of synapsin I immunostaining in the cervical spinal cord of mice with a traced hindlimb CST (BDA, red) perfused 10 days following SCI (D) and at 3 weeks post-injury (E). Yellow arrows indicate boutons that were immunoreactive for synapsin I, white arrows indicate those that were not. (D′–D′″) Single plane confocal image of the bouton boxed in D showing the collateral (D′, BDA, white), the synapsin I staining (D″; white) and the overlay (D′″; BDA, red; synapsin I, green) at 10 days after SCI. (E′–E′″) Single plane confocal image of the bouton boxed in E showing the collateral (E′; BDA, white), the bassoon staining (E″; white) and the overlay (D′″; BDA, red; synapsin I, green) at 3 weeks after SCI. (F) Quantification of the number of boutons on hindlimb CST collaterals that were immunnopositive for synapsin I at 10 days and 3 weeks following SCI in the cervical cord. The percentages were normalized to the expression pattern in the lumbar cord (L) of control animals (which was set to 100%). (G) Quantification of the co-expression of bassoon and synapsin I in boutons of CST collaterals of animals perfused at 3 weeks after injury (expressed as percentages of all immunoreactive boutons). Scale bar in A (also for B, D, E), 10 µm and in A′ (also for A″–E′″), 3 µm.

To determine the synaptic differentiation of the newly formed CST boutons we traced the hindlimb CST and then stained cervical and lumbar spinal cord sections with antibodies against two synaptic markers: bassoon, a marker of the presynaptic active zone and synapsin I, a protein that regulates neurotransmitter release at the synapse (**Fig. 4**). We first determined the percentage of boutons that are immunoreactive for the synaptic markers in the lumbar spinal cord of unlesioned mice (n = 2 mice). Of these “control” boutons 51% were immunoreactive for synapsin I and 52% were immunoreactive for bassoon. As these values likely represent the mature expression pattern, this value was set as 100% and the immunoreactivity in newly formed boutons was expressed as a percentage of the mature expression pattern. The analysis of CST boutons in the cervical spinal cord at 10 days and 3 weeks after lesion then showed that the expression of both bassoon (**Fig 4 A–C**) and synapsin I (**Fig. 4 D–F**) is low at 10 days after lesion but is comparable to the expression pattern observed in the lumbar spinal cord of unlesioned mice by 3 weeks. Double-immunostaining experiments further showed that 3 weeks after lesion 80.5±4.5% of the immunoreactive CST boutons are double positive for synapsin I and bassoon while comparably few of them showed the expression of only one marker (8±2% are only immunoreactive for synapsin I and 11.5±6.5% are only immunoreactive for bassoon, **Fig. 4 G**).

### CST collaterals refine their contacts on interneurons between 3 and 12 weeks after lesion

**Figure 5:**
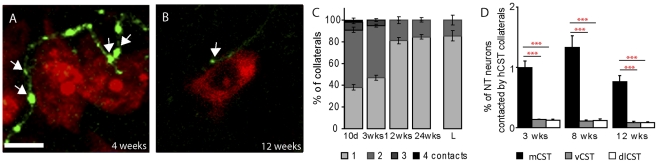
Analysis of CST contacts onto cervical interneurons after SCI. (A,B) Confocal images of contacts (arrows, defined as boutons in apposition to neuronal cell bodies) between hindlimb CST collaterals (YFP, green) and the cell bodies of cervical interneurons (Neurotrace, red) at 4 weeks (A) and 12 weeks (B) following SCI. (C) Quantification of the number of contacts a given hindlimb CST collateral makes with the cell body of a single interneuron at different timepoints after SCI as well as in the lumbar spinal cord of unlesioned animals. (D) Quantification of the percentage of Neurotrace (NT)-stained interneurons contacted by collaterals emerging from the different CST components at multiple timepoints following the lesion. Asterisks indicate significant difference compared to main CST collaterals. Scale bar in A (also for B), 15 µm.

To investigate how the projection pattern of CST collaterals evolves over time, we analyzed the number of contacts that an individual collateral formed with the cell bodies of spinal interneurons (**Fig. 5**). We first determined the mature projection pattern by evaluating contacts of hindlimb CST collaterals onto interneurons in the lumbar spinal cord. Here, we found that in most cases (85.5±4.7%, n = 2 animals and 59 collaterals) a CST collateral forms 1 and in some cases (14.5±4.7%) 2 contacts on spinal interneurons (**Fig. 5 C**). In contrast, the majority of newly emerging CST collaterals in the cervical spinal cord displayed multiple (up to 4) contacts on spinal interneurons at 10 days after lesion (**Fig. 5 C**). This “multiple contact” pattern still persisted at 3 weeks after lesion (**Fig. 5 A, C**). The mature contact pattern was only present at 12 weeks after lesion and at this time more than 80% (81.1±3.1%, n = 3 animals and 168 collaterals) of collaterals only showed 1 contact per interneuron (**Fig. 5 B, C**). The mature pattern then persisted over time and was still present at 24 weeks after injury (**Fig. 5C**). The analysis of the individual CST components showed that at 3, 8 and 12 weeks after lesion most of the contacts on interneurons were formed by collaterals emerging from the main CST tract (**Fig. 5D**).

## Discussion

**Figure 6:**
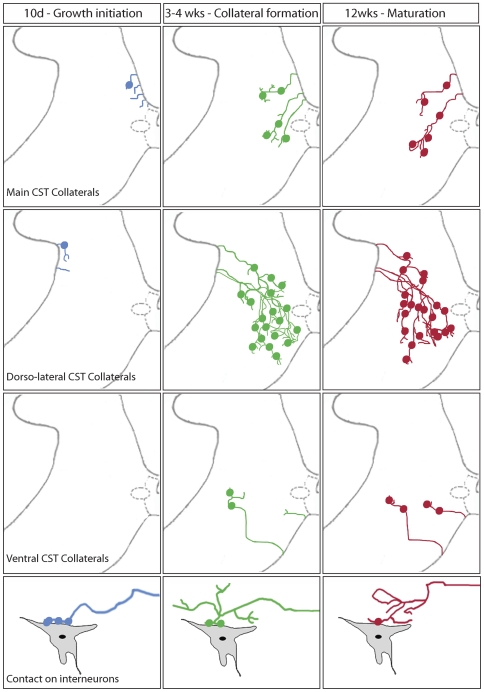
Schematic representation of hindlimb CST remodeling following SCI. Scheme illustrating the formation of cervical collaterals derived from the main CST (upper row) and the minor dorsolateral (2^nd^ row) and ventral (3^rd^ row) CST components at 10 days (blue), 3–4 weeks (green) and 12 weeks (red) after SCI. Bottom row illustrates the refinement over time of the contacts between CST collaterals and cervical interneurons.

The plastic reorganization of axonal connections is an important element of the recovery process after CNS damage. This is exemplified by the remodeling of lesioned CST fibers after spinal cord injury. Previous work has shown that the sprouting of new CST collaterals above the level of the lesion is a key step in the formation of intraspinal detour circuits that contribute to functional recovery after traumatic and inflammatory lesions of the spinal cord [6], [7], [14], [20]. Here, we can show that these collaterals form and mature in distinct phases (**Fig. 6**). In the growth initiation phase that encompasses the first 10 days after lesion, CST collaterals emerge and, at least in the case of the main and dorso-lateral CST, start to enter the cervical gray matter. In the collateral formation phase that covers the ensuing weeks, these collaterals elongate, branch and form synaptic contacts in the cervical gray matter. The final maturation phase, 12 weeks after injury, is then characterized by the small-scale refinements of the projection pattern that includes the removal of excessive inputs from interneurons. Another example of this refinement process is provided by our previous analysis [6] of CST contacts onto two distinct interneuronal populations, the long propriospinal neurons which connect the cervical spinal cord to the lumbar spinal cord [15]–[18] and the short propriospinal neurons that form intracervical connections [21], [22]. The cell bodies of these interneurons are located side by side in the cervical gray matter. Indeed, at 3 weeks after lesion - at the end of the formation phase - similar fractions of long and short propriospinal neurons are contacted by CST collaterals. However at 12 weeks - at the end of the maturation phase - many of the contacts on short propriospinal neurons have been removed while contacts on long propriospinal neurons persisted [6]. Taken together with the results of this study, it seems that the main aim of the maturation phase is the removal of excessive connections and the strengthening of pertinent connections. The emergence and selection of CST collaterals thus shows interesting parallels to the initial formation and refinement of neuronal connections in development. In the neuromuscular system it has been shown that initially exuberant connections between motor neurons and muscle fibers are formed, leading to the innervation of single neuromuscular junctions by multiple axons [23]. Over time, most of these inputs are then removed and only a single axon remains to innervate the junction [24], [25].

Similarly, during the development of the CNS initially excessive connections are formed that are later pruned. A classical example for the removal of excessive connections is the pruning of early corticospinal projections that originate from the occipital cortex [26], [27]. The refinement of CST connections during development however extends beyond this large scale pruning. For example, it is known that in different species the initial termination pattern of the CST encompasses the entire gray matter from dorsal to ventral horn and becomes more restricted later on [28]–[31]. In addition, while most corticospinal fibers in adults terminate contralateral to their origin in the cortex, during development CST axons also show extensive projections to the ipsilateral spinal gray matter. This can be at least partially explained by the findings that during development a larger proportion of axons appear to descend ipsilaterally without decussating in the pyramid while other projections cross not only in the decussation but again in the spinal cord [32]. At least some of these initial CST connections, including many of the double-crossed or uncrossed collaterals appear to be transient and are removed during the maturation of the CST [32], [33]. Further work will be necessary to determine to what extend these structural commonalities between developmental and post-injury remodeling also imply common regulatory mechanisms. For example, it will be interesting to see if neuronal activity patterns, that are important determinants of competition at the neuromuscular junction [34]–[36] or during CST development [37] also regulate the fate of newly formed CST collaterals. Likewise it will be important to explore to what extend molecules that affect pruning such as the semaphorins/plexins [38], [39], ephrins [40] or components of the wlds pathway [41] also influence the removal of CST collaterals.

The excessive formation and subsequent sorting of connections is one way how the specificity of new CST connections can be established. Another measure to ensure specificity is the targeting of axons to specific neurons or regions of the spinal cord. In the case of the newly formed CST connections, the refinement of initial connections during the maturation phase suggests that initial targeting is not established by specific recognition of single neurons. On the other hand, the distribution of newly formed CST collaterals in the spinal cord gray matter indicates that specific regions of the spinal cord, in particular the intermediate layers V–VII are preferentially targeted by CST collaterals (see **Fig. 1C**). Taken together our findings thus suggest that a combination of “region-specific” targeting that guides collaterals to the intermediate layers of the spinal cord and a subsequent refinement process that removes excessive connections collaborate to ensure specific targeting of newly formed CST connections to intraspinal relay neurons.

It should be noted however, that while most newly formed CST collaterals end in the intermediate layers of the spinal cord, some fibers reach the ventral horn and might form contacts with ventral motoneurons [42]. These direct connections of hindlimb CST axons to forelimb motoneurons could be one anatomical substrate that underlies the shift of motor maps that occurs both in animal and humans in response to spinal cord injury [43]–[45].

A second important finding of our study is that different CST components contribute to the remodeling of the CST. Anterograde tracing revealed three distinct localizations of CST fibers in the spinal cord, with the majority of fibers located in the main CST component at the bottom of the dorsal funiculus and a smaller proportion of fibers located in the dorso-lateral funiculus. Only very few fibers were observed in the ventral funiculus, contralateral to the main and dorso-lateral CST. These ventral fibers together with the fibers in the dorso-lateral funiculus form the minor CST components. This structure of the CST is in accordance with previous reports in mice, where the ventral CST component is relatively small [13], [46], [47], as well as in other rodents [11], [12], [48]–[50]. We further observed that following injury the number of fibers in the ventral component that is spared by the lesion is increased. This is probably due to sprouting of additional CST collaterals that can enter the ventral white matter tract as previously described [51]. Our analysis of collateral formation reveals both commonalities and differences between the distinct CST components. For example, the overall timecourse of collateral initiation, formation and maturation appears mostly similar in major and minor CST components. The comparably lower number of CST collaterals derived from the minor CST tracts detected in the gray matter at 10 days after injury likely does not reflect a different growth initiation but rather the longer distance between the parental axons and the gray matter border. It is interesting to note that while the overall timecourse of collateral formation is similar, the number of collaterals an individual CST axon sends to the gray matter differs substantially between the CST components. A CST axon running in the ventral funiculus, for example, extends more than 10-fold more collaterals into the gray matter at 3 weeks after lesion than a main CST axon (**Fig. 1 H, P**). These findings suggest an important role for ventral CST fibers in the CST remodeling process. As after a midthoracic lesion, the ventral funiculus consists of both pre-existing ventral fibers as well as newly sprouted collaterals likely derived from other CST components, it is possible that both unlesioned fibers and new collaterals emerging from lesioned CST fibers contribute to this response. An important role of ventral fibers is in line with previous experiments in rats that have demonstrated that ventral CST fibers can play an important role for the recovery of CST function [52]. However, it appears that not only the relative number of collaterals emerging from a given CST component but also their complexity differs between CST components. For example, collaterals emerging from the dorso-lateral CST contain several-fold more branch points and boutons at 4 weeks after lesion than collaterals emerging from main or ventral CST. This might suggest that dorso-laterals CST contact different target cell populations. The idea that distinct CST components target distinct cell populations is consistent with our previous observation that dorso-lateral CST collaterals appear to be primarily responsible for direct contacts on motoneurons in the lumbar spinal cord [13]. Taken together the characteristic differences between main and minor CST components strongly suggest that individual components might play distinct roles in the recovery process. The single collateral tracing techniques established in this study can in the future help to further define these distinct roles in different lesion paradigms. This is of interest as the remodeling process after a spinal lesion likely extends beyond the corticospinal tract to other supra- and intraspinal tract systems. For example, the reticulospinal tract has been shown to spontaneously sprout after SCI [53], [54] and the spontaneous restoration of serotonergic activity, likely mediated by the remodeling of serotonergic circuits, was found to contribute to functional recovery [55], [56].

Finally our study demonstrates that once collaterals from all CST components are formed they by and large persist long-term - in our experiments at least up to the end of the observation period (24 weeks after lesion for the population analysis and 12 weeks after lesion for the analysis of individual collaterals). Our analysis further shows that after early formation and refinement of the collaterals, very little changes to the collateral number, structure and contact pattern are observed beyond 12 weeks after lesion. This suggests that an early critical period for CST remodeling exists during which the formation or refinement of collaterals can be influenced. However after this period, newly formed connections appear to remain stable. This defines a time-window for therapeutic interventions that are likely most effective in the first 10 days after lesion if the aim is to improve collateral initiation, between 10 days and 3–4 weeks if they aim to support collateral formation and between 3–4 and 12 weeks if the aim to modulate target connections. This is of interest as despite the spontaneous remodeling of axonal connections substantial functional impairments often remain following experimental and clinical spinal cord injuries. It will therefore be important to develop therapeutic strategies that can enhance the remodeling process. One promising approach could be to foster the intrinsic neuronal growth response of cortical projection neurons targeting, for example, c-AMP and its downstream mediators [57], the growth cone-associated proteins GAP43 and CAP23 [58], components of the PTEN/mTOR pathway [59], [60] or the JAK-STAT pathway [60], [61]. Another possible way to enhance axonal remodeling is through rehabilitation. Several studies [43], [62]–[64] have already demonstrated the positive effect of rehabilitation on axonal sprouting following spinal cord injury. Care needs to be taken however not to favor task-specific rewiring at the cost of other tasks. Several studies have indeed shown that experimental rehabilitation schemes in which one task is trained repetitively will lead to improvement in this task to the detriment of other tasks [43], [65]–[67]. To our mind, the analysis techniques introduced in this study can in the future help to evaluate whether and how these therapeutic approaches can improve axonal remodeling after injury.

## Materials and Methods

### Ethics Statement

All animal experiments conformed to the institutional guidelines and were approved by the Animal Study Committee of the Regierung von Oberbayern. Approval ID: 55.2-1-54-2531-127-05.

### Animals

Adult C57BL/6 female mice 6–8 weeks old, *Thy1-Stp-YFP* and *Thy1-Brainbow* mice (line TYC9, kindly provided by J. Livet, INSERM) were used in this study. C57BL/6 mice were used for all conventional CST tracing experiments. *Thy1-Stp-YFP* mice express yellow fluorescent protein (YFP) in neurons after *Cre*-mediated excision of a floxed STOP-sequence [13], [68]. *Thy1*-*Brainbow* mice show a combinatorial expression of different fluorescent proteins after *Cre*-mediated excision of Lox sites [19]. Briefly, in the brainbow-1.0 mice used in this study, lox P sites alternate with incompatible lox variant (lox2272) sites. The *Cre recombinase* thus randomly chooses between different initial excision events. As the initial excision between a pair of identical lox sites removes one of the other pair, it prevents multiple recombination. Before *Cre* action, only the gene following the promoter is expressed (RFP). The recombination then switches expression to either YFP or M-CFP depending on the site of the initial recombination event. Further, the presence of multiple copies of the brainbow construct that recombine independently can lead to the co-expression of different fluorescent proteins [19]. *Thy1-Stp-YFP* and *Thy1-Brainbow* mice were used for single collateral analysis.

### Spinal Cord Injury

**Figure 7:**
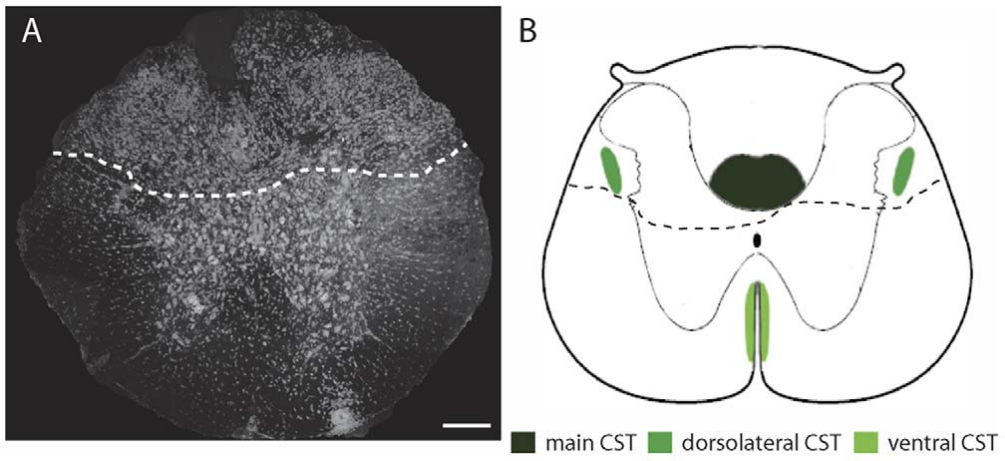
Illustration of a dorsal hemisection of the thoracic spinal cord. (A) Confocal image of a cross-section of the thoracic (T8) spinal cord of a mouse perfused 12 weeks after dorsal hemisection (counterstained with Neurotrace). Dashed line indicates lesion border. (B) Schematic representation of the location of the different CST components (highlighted in different shades of green) in relation to this lesion (outlined by dashed line from A). Scale bar in A, 200 µm.

Mice were anesthetized with a subcutaneous injection of Ketamin/Xylazin (Ketamine 150 mg/kg, Xylazine 10 mg/kg). A laminectomy was performed at thoracic level 8 (T8) and a dorsal hemisection of the spinal cord was performed with fine iridectomy scissors. This lesion interrupts the main dorsal and the minor dorso-lateral CST components but not the minor ventral CST component (**Fig. 7**). After surgery animals were heated, rehydrated and treated with analgesics (which were also administrated immediately before surgery).

### rAAV-Cre

AAV1/2-CAG-HA-NLS-Cre-WPRE-BGH-polyA expression vectors were used to generate viral particles in which the CAG promoter consists of the chicken β-actin promoter hybridized with the CMV immediate early enhancer sequence. The CAG promoter drives the expression of the P1 *Cre recombinase*, the N-terminus of which is fused to an HA-tag followed by a nuclear localization signal (NLS). The woodchuck post-transcriptional regulatory element (WPRE) and the presence of the bovine growth hormone (BGH) polyadenylation sequence ensure high transcription following transduction. AAV1/2 particles were generated by GeneDetect.com Ltd.

### Anatomical tracing of hindlimb corticospinal tract (CST)

#### Population analysis of CST collaterals

To study reorganization of both the major and minor CST components we traced the hindlimb CST in adult C57BL/6 mice by bilateral pressure injections as previously described [14]. For this purpose, 1 µl of a 10% solution of biotinylated dextran amine (BDA 10 000, Molecular Probes) was slowly injected with a glass capillary (tip diameter of about 20 µm) into lamina V of the hindlimb motor cortex (coordinates: −1.3 mm posterior to bregma, 1 mm lateral to bregma, 0.6 mm depth). The micropipette remained in place for 2 minutes after completion of the injection to minimize backflow and diffusion of the tracer.

#### Single CST collateral analysis

To study the projection pattern of individual collaterals, we first determined the amount of rAAV necessary to label single hindlimb CST collaterals by varying the injected volume. We then performed bilateral pressure injections of 0.3 µl of a rAAV-Cre (titer: 1×10^12^ genomic particles/ml) into lamina V of the hindlimb motor cortices of *Thy1-Stp-YFP* and *Thy1-Brainbow* mice. The micropipette remained in place for 2 minutes after completion of the injection to minimize backflow and diffusion of the virus.

### Tissue preparation and immunohistochemistry

Animals were perfused transcardially with 4% paraformaldehyde (PFA). Brains and spinal cords were dissected, postfixed overnight and cryoprotected in 30% sucrose for 3 days. For the population analysis of CST collaterals we analyzed the cervical spinal cord of C57BL/6 mice between the spinal level C3 and C5, where the cell bodies of long propriospinal neurons are located. For this purpose coronal sections (50 µm thickness) were cut on a vibratome and processed as described previously [69]. The hindlimb CST was revealed after BDA tracing using 0.4% ammonium nickel sulfate (Sigma), 0.015% DAB (Sigma), 0.004% H_2_O_2_ in 50 mM Tris buffer (pH 8) resulting in a black reaction product. For the analysis of individual CST collaterals consecutive coronal sections (100 µm thickness) of the cervical spinal cord of *Thy1-Stp-YFP* and *Thy1-Brainbow* mice were cut on a vibratome and mounted on gelatinized glass slides. Sections were then incubated with a rabbit anti-GFP antibody (diluted 1∶500, Invitrogen) overnight at 4°C and on the next day with a goat-anti-rabbit secondary antibody conjugated to Alexa Fluor 488 (Invitrogen). Finally, sections were counterstained with Neurotrace 435 (diluted 1∶500, Invitrogen) to identify the cell bodies of spinal interneurons.

For analysis of synaptic maturation 20 µm thick cryostat sections derived from animals, in which the hindlimb CST was labeled with BDA, were immunostained for synapsin I or bassoon as follows. Sections were incubated with ABC (Vector Laboratories) and primary polyclonal antibodies reactive against either synapsin I (Millipore, 1∶500) or bassoon (Synaptic System, 1∶500) in Tris buffer containing 0.3% Triton X-100 (Sigma) and 2.5% goat serum serum (Invitrogen) overnight at 4°C. For double immunostaining the polyclonal anti-synapsin I antibody (dilution same as above) was combined with a mouse monoclonal anti-bassoon antibody (dilution 1∶100, Enzo Life Sciences). After a 20 min tyramide amplification (Biotin-XX, TSA Kit #21, Invitrogen) to detect BDA, the sections were incubated overnight with Streptavidin conjugated to Alexa Fluor 594 (1∶500, Invitrogen) and a goat-anti-rabbit antibody conjugated to Alexa Fluor 488 (1∶500, Invitrogen). Counterstaining was performed using NeuroTrace 435 (1∶500, Invitrogen) and sections were mounted in Vectashield (Vector Laboratories).

### Quantification of anatomical reorganization

#### Population analysis of CST collaterals

Fibers exiting from main and minor CST components and entering the grey matter were counted in 30 consecutive coronal sections of the cervical spinal cord using a IX71 microscope (Olympus) with a ×40 (NA 0.65) objective. To correct for inter-animal differences in tracing efficiency, the number of CST collaterals was divided by the number of traced fibers in the respective CST component and expressed as a ratio of collaterals per CST fiber.

#### Single collateral analysis

Consecutive coronal sections of the cervical spinal cord of *Thy1-Stp-YFP* and *Thy1-Brainbow* mice were imaged on an Olympus FV1000 confocal microscope. Image stacks were acquired with a ×20 oil objective and processed using ImageJ (http://rsbweb.nih.gov/ij/) and Adobe Photoshop software. Alignment and tracing of collaterals in consecutive sections was performed in Adobe Photoshop. Collateral properties (collateral length, number of branch points) were measured using the NeuronJ plugin in ImageJ.

#### Contacts on interneuronal cell bodies

To quantify the contacts onto interneuronal cell bodies 20 µm sections of the cervical spinal were scanned with a ×20 (NA 0.85) oil immersion objective. Single hindlimb CST collaterals (labelled with BDA) were followed and the number of boutons in contact with the cell body of an interneuron (labelled with Neurotrace) was counted.

#### Expression of synaptic markers

To determine the percentage of boutons that express the synaptic markers synapsin I and bassoon image stacks were acquired with an Olympus FV1000 confocal microscope equipped with standard filter sets and a ×60 (NA 1.45) oil immersion objective. Tissue from the population analysis was used for this analysis as the high number of BDA-labelled collaterals in this tissue allowed us to analyse sufficiently large numbers of CST boutons. The total number of boutons as well as the number of these boutons that expressed synapsin I or bassoon were counted. To analyze the co-expression of synapsin I and bassoon, sections from the cervical spinal cord of animals perfused 3 weeks after lesion (n = 3 mice) were used. The number of CST boutons immunoreactive for either bassoon, synapsin I or both was determined and expressed as percentage of all immunoreactive boutons. All quantifications were performed by a blinded observer.

### Image processing

Image stacks obtained with confocal microscopy were processed using ImageJ software to generate maximum intensity projections. To obtain final images, these maximum intensity projections were processed in Adobe Photoshop using gamma adjustments to enhance visibility of intermediate gray values and median filtering to suppress noise when necessary. For the representation of CST collaterals (**Fig. 1 A–C**) 5 consecutive sections were reconstructed and overlaid.

### Statistical evaluation

Results are given as mean ± SEM unless indicated otherwise. For paired comparison data were analyzed by the Student's *t* test. For multiple comparisons a two-way ANOVA followed by a Tukey's or a Bonferroni *post hoc* was performed using Graphpad Prism 5.01 for Windows (GraphPad Software). Significance levels are indicated as follows: *p<0.05; **p<0.01; ***p<0.001.
